# Investigation of the association between coffee and risk of RA—results from the Swedish EIRA study

**DOI:** 10.1186/s13075-022-02862-2

**Published:** 2022-07-26

**Authors:** Helga Westerlind, Justine Dukuzimana, Xiaomin Lu, Lars Alfredsson, Lars Klareskog, Daniela Di Giuseppe

**Affiliations:** 1 Clinical Epidemiology Division, Department of Medicine, Karolinska Institutet, Solna, Sweden; 2 Institute of Environmental Medicine, Karolinska Institutet, Solna, Stockholm Sweden; 3 Rheumatology Division, Department of Medicine (Solna), Karolinska Institutet, Solna, Sweden

**Keywords:** Rheumatoid arthritis, Risk, Coffee, Diet, ACPA, Smoking, Rheumatoid factor, Epidemiology

## Abstract

**Background:**

Studies on the association between coffee, a modifiable lifestyle factor, and rheumatoid arthritis (RA), a chronic autoimmune disease primarily affecting the joints, have been conflicting. The aim of the present study was to study the association between coffee consumption and risk of RA in the context of different lifestyle factors.

**Methods:**

We included 2184 cases (72% women, mean age 55 years) newly diagnosed with RA during 2005–2018 in Sweden and 4201 controls matched on age, sex, and residential area. Data on coffee consumption was collected through a food frequency questionnaire and categorized into < 2 (reference), 2–< 4, 4–< 6, and ≥ 6 cups/day. We calculated odds ratios (OR) with 95% confidence intervals (CI) for coffee consumption and risk of RA, in a crude model (taking matching factors into account), and then adjusted first for smoking and further for BMI, educational level, alcohol consumption, and physical activity. We also stratified analyses on sex, smoking, rheumatoid factor, and anti-CCP2 status.

**Results:**

In the crude model, high coffee consumption was associated with increased risk of RA (OR = 1.50, 95% CI 1.20–1.88 for ≥ 6 cups/day compared to < 2 cups). After adjusting for smoking, the OR decreased and was no longer statistically significant (OR = 1.16, 95% CI 0.92–1.46) and decreased further in the full model (OR = 1.14 95% CI 0.89–1.45). This pattern held true in all strata.

**Conclusion:**

The findings from this large, population-based case-control study did not support a significant association between coffee consumption and risk of RA as a whole nor within different subgroups.

## Introduction

For rheumatoid arthritis (RA), a chronic disease affecting the joints of the body, genetic risk factors have long been established and the heritability is estimated to be around 40% [1]. Among environmental and lifestyle factors, smoking is a well-established risk factor with evidence from multiple studies, while for many other factors the evidence is still inconclusive [2, 3]. Identification of modifiable risk factors, like diet, is of importance as they would allow implementation of personalized and stratified primary prevention with specific, targeted interventions among high risk individuals. However, diet and lifestyle factors are complex with strong interdependence, and it is therefore important to not study a factor on its own but also take potential confounding factors into consideration. Coffee, one of the world’s most popular beverages, is indeed one of these complex factors, and it is long known that cigarette smoking and coffee consumption are highly correlated [4].

Some studies have been devoted to the study of coffee consumption and risk for RA and a meta-analysis from 2014 that included data from five different studies across the world on coffee consumption and risk of RA concluded that coffee is a risk factor for RA ([5], with later published Errata, in which the effect of coffee drastically decreased). However, the results from this meta-analysis, often cited, is questionable, as the studies driving the association did not adjust for smoking [6, 7], while those studies in the meta-analysis that did adjust for smoking, did not find any association [8–10]. Later studies, adjusting for smoking, also did not observe an association between coffee consumption and risk of RA [11].

The association between environmental risk factors has been reported to differ between seropositive and seronegative RA [12, 13]. Also for coffee consumption the data is conflicting in relation to these RA-subtypes. Of four studies investigating the association between coffee consumption and risk of seropositive RA, three reported significant associations [7, 9, 14], while one did not [8]. These four studies all adjusted for smoking. For seronegative RA, two studies, both including smoking in their models, have reported no association to coffee [9, 14]. Further, some of the studies on coffee consumption and risk of RA have been performed in cohorts consisting of only women [8, 9, 11].

Despite these previous studies, uncertainty still remains concerning the association between coffee consumption and risk for RA, as the studies so far have been quite small and have, in some cases, not adjusted for important environmental/lifestyle factors such as BMI, physical activity, and smoking. We therefore believe that there is a need for additional analyses. The overall goal of this project was to study the association between coffee consumption and risk of RA, in the context of different lifestyle factors. For this purpose, we used the Epidemiological Investigation of RA (EIRA) case control study which encompasses more than twice the number of cases and controls as compared with the entire previous meta-analysis, which also has access to information on a number of additional potential confounders.

## Materials and methods

We included individuals from EIRA recruited from October 2005 until May 2018. EIRA is a population-based case-control study including patients newly diagnosed with RA according to the American College of Rheumatology (ACR) 1987 criteria or the 2010 ACR/European League Against Rheumatism (EULAR) classification criteria for RA [15], from rheumatology clinics in the southern and middle parts of Sweden. For every case, two controls, matched on age, sex, and residential area, are randomly selected. Upon inclusion and consenting to participating, both cases and controls are asked to donate blood and answer a questionnaire on environment and lifestyle habits, such as diet. EIRA has been extensively described elsewhere [16, 17]. In the present study, we included cases and controls that had filled out the 124-item food frequency questionnaire (FFQ) included in the EIRA questionnaire. The questionnaire including the FFQ was sent out to 2602 cases and 6825 controls. Of these 2370 cases and 4701 controls chose to participate, making the participation rate 91% among the cases and 69% among the controls. We further excluded participants that did not consent or fill out the FFQ. Cases without at least one control and controls without a corresponding case were also excluded from the analysis. From EIRA, we collected data on age, sex, anti-CCP2, and rheumatoid factor (RF) status and several lifestyle factors (described below). Anti-CCP2 status was determined using the commercial assay from Eurodiagnostica, using > = 25 arbitrary units as cutoff for positivity and RF status was determined by the diagnosing rheumatologist.

### Coffee consumption

In the FFQ, participants were asked to provide information on either daily or weekly consumption of brewed and cooked coffee the year before RA diagnosis. We summarized the total amount of coffee intake to calculate the average daily coffee consumption and categorized this into < 2 cups/day, 2–< 4 cups/day, 4–< 6 cups/day and ≥ 6 cups/day. Individuals with no information on coffee consumption were assumed to not drink coffee according to the zero-consumption assumption [18].

### Covariates

To take information on lifestyle into account, we included data on cigarette smoking, body mass index (BMI), educational level, alcohol consumption, tea consumption, and physical activity. Cigarette smoking was categorized into never, former, current, non-regular and those smoking other forms of tobacco, with never smoker used as reference. BMI was divided into low (< 18.5kg/m^2^), normal (18.5kg/m^2^–< 25 kg/m^2^), overweight (≥ 25 kg/m^2^–< 30 kg/m^2^), and obese (> 30 kg/m^2^) with normal BMI serving as reference category. Educational level was divided into < 10 years of education, 10–12 years of education, and > 12 years of education with 10–12 years of education used as reference. Total daily consumption of alcohol was categorized into 0–14 gr/day, 14–40 gr/day, 41–88 gr/day, and > 88 gr/day with 0–14 gr/day as reference, with 14 grams of alcohol being approximately equivalent to one drink. Tea consumption was categorized into no consumption, > 0–< 1 cups/day, ≥ 1–< 2 cups/day, and ≥ 2 cups per day, in analogy with our previous study [19]. Finally, data on physical activity was estimated based on reported level of physical activity 5 years before and the year before inclusion in EIRA. We combined the data on physical activity into always sedentary (those who reported no or moderate exercise at both time points, used as reference), always active (moderate regular or regular exercise at both time points), decreasing activity (moderate regular or regular activity five years before, but no or moderate exercise the year before), and increasing activity (no or moderate exercise 5 years before, moderate regular or regular exercise the year before).

### Statistical analysis

We calculated mean and proportion as appropriate for the demographic data and stratified on case status and level of coffee consumption. We then ran a conditional logistic regression and estimated odds ratios (OR) and 95% confidence intervals (CI) for the crude association between coffee consumption and RA status taking the matching factors into consideration. We first adjusted the model for smoking and then further for BMI, educational level, alcohol consumption, tea consumption, and physical activity. We tested for a linear trend by using the median value for each exposure category. We also performed analyses stratified on sex, smoking (never/ever smoker), anti-CCP2, and RF status. For the analyses stratified on smoking and anti-CCP2 status, we used unconditional logistic regression where in the crude model we adjusted for age, sex, and residential area.

### Sensitivity analysis

To test the robustness of the results, we performed two sensitivity analyses: one in which we excluded all individuals that did not report any coffee consumption and one where we split the lowest consumption group into never drinker and > 0–< 2 cups/day. For the latter analysis, we used the never drinkers as reference to contrast the potential risk of coffee consumption against not drinking coffee.

## Results

We included 2184 (*n* = 1561 (72%) women) cases and 4201 (*n* = 3004 (72%) women) controls in the study. The mean age was, because of the matched design, 55 years (SD 14) among both cases and controls and 1471 (68%) of the cases were anti-CCP2 positive. For both cases and controls, women had lower coffee consumption compared to men, and the proportion of tea consumers was lowest among those that consumed the highest amount of coffee. The mean age was lowest among those drinking < 2 cups/day for both cases and controls. The proportion of current smokers increased with coffee consumption (Table 1).

**Table 1:** Demographics of 2184 newly diagnosed RA cases and 4201 controls from the EIRA study, stratified by coffee consumption

	RA cases				Controls			
Coffee, cups/day	< 2 cups/day	2–< 4 cup/day	4–< 6 cups/day	≥ 6 cups/day	< 2 cups/day	2–< 4 cup/day	4–< 6 cups/day	≥ 6 cups/day
Coffee, median cups/day	0.4	2.5	4.0	7.0	0.3	2.4	4.0	6.4
***N***	571	947	483	183	1164	1874	907	256
**Female,** ***n*** **(%)**	445 (78)	684 (72)	327 (68)	105 (57)	894 (77)	1401 (75)	573 (63)	136 (53)
**Age, mean (SD)**	49.8 (16.7)	56.4 (13.0)	56.4 (11.1)	55.5 (9.7)	51.1 (16.0)	56.6 (13.0)	56.2 (11.3)	55.2 (11.4)
**Tea,** ***n*** **(%)**								
**0 cups/day**	149 (26)	377 (40)	289 (60)	120 (66)	309 (27)	765 (41)	505 (56)	182 (71)
**≤ 2 cups/day**	116 (20)	211 (22)	84 (17)	35 (19)	198 (17)	368 (20)	155 (17)	34 (13)
**2–3 cups/day**	110 (19)	175 (19)	73 (15)	16 (9)	219 (19)	376 (20)	137 (15)	23 (9)
**> 3 cups/day**	196 (34)	184 (19)	37 (8)	12 (7)	438 (38)	365 (20)	110 (12)	17 (7)
**Smoking,** ***n*** **(%)**								
**Current**	68 (12)	168 (18)	159 (33)	87 (48)	76 (7)	207 (11)	196 (22)	94 (37)
**Never**	297 (52)	338 (36)	106 (22)	34 (19)	689 (59)	946 (51)	360 (40)	75 (29)
**Former**	145 (25)	347 (37)	174 (36)	52 (28)	264 (23)	536 (29)	272 (30)	61 (24)
**Non-regular**	43 (8)	62 (7)	27 (6)	3 (2)	83 (7)	117 (6)	39 (4)	13 (5)
**Other**	20 (2)	25 (3)	11 (2)	6 (3)	29 (3)	47 (3)	29 (3)	10 (4)
**Education,** ***n*** **(%)**								
**< 10 years**	95 (17)	187 (20)	121 (25)	47 (26)	133 (11)	284 (15)	174 (19)	55 (22)
**10–12 years**	166 (29)	201 (21)	111 (23)	58 (32)	298 (26)	443 (24)	243 (27)	79 (31)
**> 12 years**	310 (54)	559 (59)	251 (52)	78 (43)	722 (63)	1147 (61)	490 (54)	122 (48)
**BMI, mean (SD)**	25.7 (5.1)	25.8 (4.6)	25.8 (4.4)	26.0 (4.4)	25.3 (4.7)	25.3 (5.5)	25.6 (4.0)	25.9 (3.9)
**Alcohol intake, median grams/week (IQR)**	29.0 (61)	38.0 (69.8)	41.0 (72.0)	41.5 (63.3)	29.0 (70.0)	47.0 (76.0)	48.0 (79.0)	43.5 (93.0)

The crude ORs increased with level of coffee consumption and were for 2–< 4 cups/day 1.04 (95% CI 0.91–1.18), 4–< 6 cups/day 1.11 (95% CI 0.95–1.29), and for ≥ 6 cups/day 1.50 (95% CI 1.20–1.88), *p*-value for trend 0.0013, as compared to < 2 cups/day. When adjusting for smoking the OR decreased for all groups and lost statistical significance for the group drinking ≥ 6 cups/day (OR=1.16, 95% CI 0.92–1.46, *p*-value for trend 0.61) and in the full model, the OR for ≥ 6 cups/day was 1.14 (95% CI 0.89–1.45, *p*-value for trend 0.64) (Table 2).

**Table 2:** Odds ratios (OR) for coffee consumption and risk of developing RA among 2184 newly diagnosed RA cases and 4201 controls, matched on age, sex, and residential area

Coffee, cups/day	< 2 cups/day	2–< 4 cup/day	4–< 6 cups/day	≥ 6 cups/day	
	**Main analysis**				
*N* cases/controls	571/1164	947/1874	483/907	183/256	*p*-value for trend
Crude OR	Ref	1.04 (0.91–1.18)	1.11 (0.95–1.29)	1.50 (1.20–1.88)	0.0013
OR adjusted for smoking	Ref	0.97 (0.85–1.11)	0.93 (0.79–1.09)	1.16 (0.92–1.46)	0.61
Full model^a^	Ref	0.99 (0.86–1.13)	0.94 (0.79–1.12)	1.14 (0.89–1.45)	0.64
	**Women**				
*N* cases/controls	445/894	684/1401	327/573	105/136	
Crude OR	Ref	0.99 (0.85–1.15)	1.17 (0.97–1.40)	1.60 (1.19–2.13)	0.0022
OR adjusted for smoking	Ref	0.92 (0.79–1.07)	0.97 (0.80–1.18)	1.19 (0.88–1.61)	0.56
Full model^b^	Ref	0.95 (0.81–1.12)	1.02 (0.83–1.25)	1.27 (0.93–1.75)	0.26
	**Men**				
*N* cases/controls	126/270	263/473	153/334	78/120	
Crude OR	Ref	1.21 (0.93–1.57)	1.01 (0.75–1.35)	1.42 (0.98–2.05)	0.18
OR adjusted for smoking	Ref	1.17 (0.90–1.53)	0.91 (0.67–1.23)	1.18 (0.81–1.74)	0.79
Full model^b^	Ref	1.11 (0.84–1.47)	0.85 (0.61–1.17)	0.99 (0.66–1.48)	0.55
	**Anti-CCP2 positive**				
*N* cases/controls	384/1164	631/1874	332/907	125/256	
Crude OR	Ref	1.07 (0.92–1.24)	1.18 (0.99–1.40)	1.58 (1.23–2.02)	0.00032
OR adjusted for smoking	Ref	0.98 (0.84–1.14)	0.95 (0.79–1.13)	1.13 (0.87–1.46)	0.66
Full model^b^	Ref	0.99 (0.84–1.16)	0.96 (0.80–1.16)	1.10 (0.84–1.44)	0.72
	**Anti-CCP2 negative**				
*N* cases/controls	184/1164	313/1874	147/907	57/256	
Crude OR	Ref	1.01 (0.83–1.23)	0.97 (0.77–1.23)	1.32 (0.94–1.83)	0.28
OR adjusted for smoking	Ref	0.97 (0.80–1.19)	0.88 (0.69–1.12)	1.14 (0.81–1.59)	0.94
Full model^b^	Ref	0.99 (0.80–1.22)	0.90 (0.70–1.17)	1.14 (0.80–1.61)	0.84
	**Rheumatoid factor positive**				
*N* cases/controls	355/1164	607/1874	314/907	121/256	
Crude OR	Ref	1.09 (0.94–1.27)	1.18 (0.98–1.41)	1.61 (1.25–2.07)	0.00029
OR adjusted for smoking	Ref	1.00 (0.85–1.17)	0.94 (0.78–1.13)	1.13 (0.87–1.47)	0.71
Full model^b^	Ref	1.01 (0.86–1.18)	0.94 (0.78–1.14)	1.10 (0.84–1.45)	0.80
	**Rheumatoid factor negative**				
*N* cases/controls	209/1164	328/1874	167/907	60/256	
Crude OR	Ref	0.97 (0.80–1.17)	1.01 (0.81–1.27)	1.29 (0.93–1.76)	0.23
OR adjusted for smoking	Ref	0.93 (0.77–1.13)	0.93 (0.74–1.16)	1.13 (0.80–1.56)	0.82
Full model^b^	Ref	0.96 (0.79–1.18)	1.00 (0.78–1.27)	1.16 (0.82–1.63)	0.52
	**Never smokers**				
*N* cases/controls	297/689	338/946	106/360	34/75	
Crude OR	Ref	0.87 (0.72–1.05)	0.73 (0.56–0.95)	1.13 (0.72–1.73)	0.21
OR adjusted for smoking	N/A	N/A	N/A	N/A	N/A
Full model^b^	Ref	0.93 (0.76–1.13)	0.78 (0.59–1.03)	1.18 (0.74–1.84)	0.54
	**Ever smokers (adjusted for current smoking status)**				
*N* cases/controls	266/452	602/907	371/536	148/178	
Crude OR	Ref	1.13 (0.94–1.36)	1.18 (0.96–1.45)	1.41 (1.08–1.85)	0.01
OR adjusted for smoking	Ref	1.11 (0.92–1.33)	1.10 (0.89–1.35)	1.25 (0.95–1.65)	0.13
Full model^b^	Ref	1.08 (0.89–1.31)	1.06 (0.86–1.32)	1.19 (0.89–1.58)	0.29

^a^Adjusted for smoking, BMI, alcohol consumption, educational level, tea consumption, and physical activity

^b^Adjusted for age, sex, residential area, smoking, BMI, alcohol consumption, educational level, tea consumption, and physical activity

After stratification, the OR for risk of RA among those drinking ≥ 6 cups/day was significantly increased in the crude model among women, anti-CCP2 positive and RF positive, and smokers, but lost significance when adjusting for smoking. For men and among anti-CCP2 negative and RF negative, the same pattern was observed, although the crude OR was not significant, possibly due to the smaller sample size.

### Sensitivity analysis

As sensitivity analyses, we changed the reference group and used no-consumption of coffee as reference instead of < 2 cups/day (including also the imputed data): the ORs increased slightly, but again, were non-significant upon adjusting for smoking (Table 3). In a second sensitivity analyses, we wanted to understand the influence of the zero-assumption imputation on the results. We therefore excluded 763 individuals (12%) that had not filled out data on coffee consumption, and we observed the same pattern and magnitude of ORs as in the full cohort.

**Table 3:** Odds ratios (OR) for coffee consumption and risk of developing RA among 2184 newly diagnosed RA cases and 4201 controls, matched on age, sex, and residential area, according to sensitivity analyses

Coffee, cups/day	< 2 cups/day		2–< 4 cup/day	4–< 6 cups/day	≥ 6 cups/day
	**Removing missing coffee data**				
*N* cases/controls	329/643		947/1874	483/907	183/256
Crude OR	Ref		1.00 (0.85–1.17)	1.10 (0.91–1.32)	1.45 (1.13–1.85)
OR adjusted for smoking	Ref		0.93 (0.79–1.09)	0.92 (0.76–1.12)	1.11 (0.86–1.44)
Full model^a^	Ref		0.92 (0.77–1.09)	0.91 (0.75–1.12)	1.06 (0.81–1.39)
	**Changing reference group**				
	**0 cups/day**	**> 0–< 2 cups/day**	**2–< 4 cup/day**	**4–< 6 cups/day**	**≥ 6 cups/day**
*N* cases/controls	252/544	319/620	947/1874	483/907	183/256
Crude OR	Ref	1.13 (0.92–1.38)	1.11 (0.93–1.32)	1.18 (0.97–1.44)	1.60 (1.24–2.06)
OR adjusted for smoking	Ref	1.12 (0.91–1.38)	1.04 (0.87–1.24)	1.00 (0.82–1.22)	1.23 (0.95–1.60)
Full model^a^	Ref	1.17 (0.941–45)	1.08 (0.89–1.30)	1.03 (0.83–1.27)	1.24 (0.94–1.63)

^a^Adjusted for smoking, BMI, alcohol consumption, educational level, tea consumption, and physical activity

## Discussion

In this study, we investigated the association between coffee consumption and risk of RA in a group of Swedish newly diagnosed RA cases with matched controls. The study design allowed us to adjust for and investigate this association in the context of a magnitude of environmental and lifestyle factors. We observed a crude positive association between coffee consumption and risk of RA. However, when adjusting for smoking, the OR decreased and lost its statistical significance, and this loss was maintained and further attenuated when adjusting for a series of additional lifestyle and environmental factors previously shown to be associated with risk for RA. This pattern was consistent across all strata and in the sensitivity analyses.

The results from this large case-control study with adjustments for smoking as well as other potentially confounding environmental/lifestyle-factors are thus in line with previous studies on coffee and risk of RA that have adjusted for smoking. No previous study has, however, adjusted for the many additional lifestyle confounders analyzed here, that however have only slightly further decreased the estimates.

Previous studies that reported an association between coffee consumption and risk of RA did not adjust for smoking [6, 7]. However, since smoking is a major risk factor for RA, and strongly associated with coffee, it is likely those studies have biased results due to the lack of smoking adjustment. The importance of smoking when analyzing coffee as an exposure has been also highlighted in other contexts [20].

An additional point addressed only in some of the previous studies is that the association to environmental risk factors can differ between subsets of RA. Indeed, coffee has in some studies, after adjusting for smoking, been reported to be associated only with seropositive RA [7, 9, 14], while others have not observed this association [8]. It should however be noted that one of these studies only investigated decaffeinated coffee and risk of seropositive RA [9]. In three of these four studies, seropositivity was defined as RF positive RA [7–9], and in the fourth it was defined as anti-CCP2 positivity [14]. In the present study, we investigated the association between coffee consumption and anti-CCP2 status as well as RF status. We could however not detect any significant association confined to any of the antibody positive groups.

We could not find any association between coffee consumption and risk of RA when stratifying on sex. These results replicate the previous studies performed in women only [8, 9, 11] and extend these studies to men and RA overall. We further extend previous studies on coffee consumption and risk of RA by stratifying on smoking, where we could not detect any association confined to never or ever smokers.

Strengths of this study include a large, well-characterized population-based case-control study which enabled us to take many different lifestyle factors into account. The data on smoking behavior was collected at diagnosis and at the same time as coffee consumption which made it possible to adjust for current smoking in the analysis among ever smokers.

Limitations include the low proportion of non-drinkers of coffee, which prevented us from using non-coffee drinkers as a reference group. We did however address this problem in a sensitivity analysis and observed the same result pattern. When we used never coffee consumer as reference group, there was potentially a slightly stronger effect observed, but as the definition of never coffee drinkers could be somewhat biased due to the zero-consumption assumption, we cannot draw any conclusions from this analysis. Further, the data did not allow to investigate the association between consumption of caffeinated and decaffeinated coffee separately, or the total caffeine intake, as we simply did not have data on this. We could also not rule out that the OR for the highest category of coffee consumption was attenuated due to non-differential misclassification of coffee consumption. However, as mentioned above, the EIRA questionnaire is sent out and filled in at the time of diagnosis, asking for coffee consumption during the previous year, which provides little time to change behavior. We have also previously reported, in a similar cohort, that coffee consumption is stable over time among women diagnosed with RA [21] and thus believe the potential misclassification to be very small. Since the questionnaire was self-reported, we cannot rule out measurement error both for coffee and other covariates. Another limitation is that this study investigates the association between self-reported coffee consumption and risk of RA, and any causal relationship between the two has not been addressed.

## Conclusions

In this big case control study with access to a large amount of environmental and lifestyle data, we found that the association between coffee consumption and risk for RA observed in a crude, non-adjusted analysis was mainly due to a co-occurrence of coffee consumption and smoking. Taking all these factors into account, we conclude that coffee consumption per se is not associated to the risk of developing RA.

## Data Availability

Due to the content of the ethical approval and consents, data on the individual level from EIRA cannot be publicly shared. For access to data on additional subsets of EIRA, please contact the principal investigators for data requests for applicable studies. For further information, go to: http://www.eirasweden.se/Kontakt_EIRA.htm
